# Identification of new reference genes with stable expression patterns for cell cycle experiments in human leukemia cell lines

**DOI:** 10.1038/s41598-024-84802-5

**Published:** 2025-01-07

**Authors:** Otília Tóth, Gergely Attila Rácz, Eszter Oláh, Máté Tóth, Edit Szabó, György Várady, Beáta G. Vértessy, Nikolett Nagy

**Affiliations:** 1 Department of Applied Biotechnology and Food Science, Faculty of Chemical Technology and Biotechnology, BME Budapest University of Technology and Economics, Budapest, Hungary; 2 Institute of Molecular Life Sciences, HUN-REN Research Centre for Natural Sciences, Budapest, Hungary; 3 Doctoral School of Biology, Institute of Biology, ELTE Eötvös Loránd University, Budapest, Hungary

**Keywords:** Molecular biology, Gene expression analysis, Reverse transcription polymerase chain reaction

## Abstract

Cell cycle-dependent gene expression analysis is particularly important as numerous genes show tightly regulated expression patterns at different phases of the cell cycle. For cancer cells, analysis of cell cycle-related events is of paramount significance since tumorigenesis is characteristically coupled to cell cycle perturbations. RT-qPCR is a highly sensitive technique to investigate cell cycle-dependent transcriptional regulation. However, for reliable evaluation of qPCR data reference genes with stable mRNA expression are required. Although several studies investigating cell cycle-dependent gene expression employ frequently used reference genes, the suitability of these reference genes has not been thoroughly investigated so far. Moreover, such potential reference genes for cell cycle analysis have not been described in the literature. Therefore, we aimed to identify reference genes characterized with stable expression throughout the cell cycle in MOLT4 and U937 human leukemia cell lines synchronized with RO-3306 CDK1 inhibitor using RT-qPCR. Here we show that for cell cycle-dependent gene expression analysis the commonly used *TBP* is suitable, while the recently recognized reference genes *SNW1* and *CNOT4* are applicable in a cell line-dependent manner. We also suggest that proper selection of reference genes for each experimental condition is crucial for reliable normalization as these aspects can severely compromise conclusions.

**Supplementary Information:**

The online version contains supplementary material available at 10.1038/s41598-024-84802-5.

## Introduction

Reverse transcription coupled quantitative PCR (RT-qPCR) is a widely utilized technique in molecular biology for the precise quantification of mRNA expression levels in cells and tissues^1–5^. Suitable reference genes characterized with low variation are required for the reliable evaluation of gene expression results^6–11^. Although RT-qPCR is a routinely used method, proper normalization generally receives little consideration, as commonly used reference genes are often applied without verifying their stability^12^. However, it has become evident that expression patterns of several frequently used reference genes highly depend on the given experimental conditions^8,13–15^, questioning their suitability in the experimental setups.

Investigation of the cell cycle is of utmost importance, as expression of several genes and proteins are under cell cycle regulation^16^. However, there is a lack of studies focusing on identifying reliable reference genes for cell cycle-dependent gene expression analysis. For normalization of gene expression data two or more reference genes characterized with low variation are needed^17,18^, however, most studies investigating cell cycle-dependent gene expression use only one reference, generally either *GAPDH*^19–22^ or *ACTB*^23,24^ for normalization.

Beyond the general issue of relying on a single, not fully validated reference gene, there are also numerous considerations to be taken into account for designing reliable cell-cycle studies. In particular, a variety of different methods have been described for synchronization of eukaryotic cells to investigate the cell cycle, such as serum starvation, contact inhibition, double thymidine block or inhibition of cyclin-dependent kinases (CDKs)^25–32^. However, most of the synchronization methods have limitations. Several studies apply combination of different synchronization methods to arrest cells in specific phases of the cell cycle^20,33,34^. Upon serum starvation cells exit into quiescent G0 phase, however, cells do not enter into G1 phase upon serum stimulation at the same rate, since this process is affected by the preceding cell growth and division state^35^. Contact inhibition is a widely used technique to arrest cells in early G1 phase, however, transformed and cancer cells often exhibit resistance, besides, it is only applicable to adherent cell lines^25^. Double thymidine block is used to synchronize cells is G1/S phase, as high thymidine concentration causes imbalance in the dNTP pool and can inhibit DNA synthesis, although reaction of cells after treatment can substantially vary depending on the particular cell type^36^. In case of using combined synchronization methods, each technique superimposes specific changes in the transcriptome and proteome^37^. A simple strategy for arresting cells in G2/M phase is the reversible inhibition of CDK1 using RO-3306^31^. When finding a suitable synchronization method for a chosen experimental design the applicability to different cell lines, optimization of treatment conditions, efficacy of particular protocols and possible negative effects on cell metabolism should be taken into account^36^.

Recognizing the lack of meticulous and reliable data for these issues, in the present study we investigated 12 candidate reference genes (*ACTB*,* CNOT4*,* GAPDH*,* HNRNPL*,* IPO8*,* PCBP1*,* PPIA*,* PUM1*,* RPL30*,* SNW1*,* TBP*,* UBC*) for cell cycle experiments. We analyzed U937 and MOLT4 human cancer cell lines, which are widely used for advancing knowledge in leukemia, immune responses and drug development^38,39^. Investigated candidate reference genes were selected based on publications available in PubMed archive, where *ACTB*,* GAPDH*,* IPO8*,* PPIA*,* PUM1*,* RPL30*,* TBP* and *UBC* were the most commonly used reference genes. Moreover, the recently recognized novel reference genes, *SNW1* and *CNOT4* were included in our study due to their high stability as indicated by the lowest coefficient variation (CV) values according to the RNA HPA cell line gene dataset of The Human Protein Atlas^40^. Furthermore, *HNRNPL* and *PCBP1* were identified as the most stable reference genes according to a study based on an analysis of large-scale expression data from The Cancer Genome Atlas (TCGA) database^41^, thus we aimed to investigate these genes as well. Regarding cell cycle dependency of candidate reference genes there are no information available in the Cell Cycle-Dependent Transcript Database of Human Protein Atlas^42^, suggesting that their expression may not be influenced by the cell cycle. Moreover, Cyclebase 3.0 database^43^ indicated that all investigated genes are non-periodic, except *PCBP1*, which has elevated expression in G2 phase, and *GAPDH* and *ACTB*, which have no data available. Although these databases do not consider these genes cell cycle dependent, targetgenereg.org dataset^44^ contains inconsistent information or suggests cell cycle dependency. Due to contradictory data available in the literature about the cell cycle dependency of candidate reference genes, we further investigated the expression of these genes during the cell cycle. With regard to the proper choice of cell cycle control, we designed a highly robust method applicable for the analysis of cell cycle-dependent gene expression using RO-3306 to synchronize cells in G2/M phase. Evaluation of gene expression data was carried out using the Comparative ΔCt method as well as NormFinder, geNorm and BestKeeper tools. We found that different reference genes are suitable for U937 alone, for MOLT4 alone and for both cell lines together based on the comprehensive ranking of four evaluation methods. We concluded that *CNOT4* was the most stable reference gene in MOLT4 cell line, whereas *SNW1* was the most stable reference gene in U937 and in both cell lines. Overall, the recently identified *SNW1* and *CNOT4* reference genes are not only applicable for gene expression normalization between different cell lines, but also for cell cycle analysis.

## Results and discussion

**Fig. 1:**
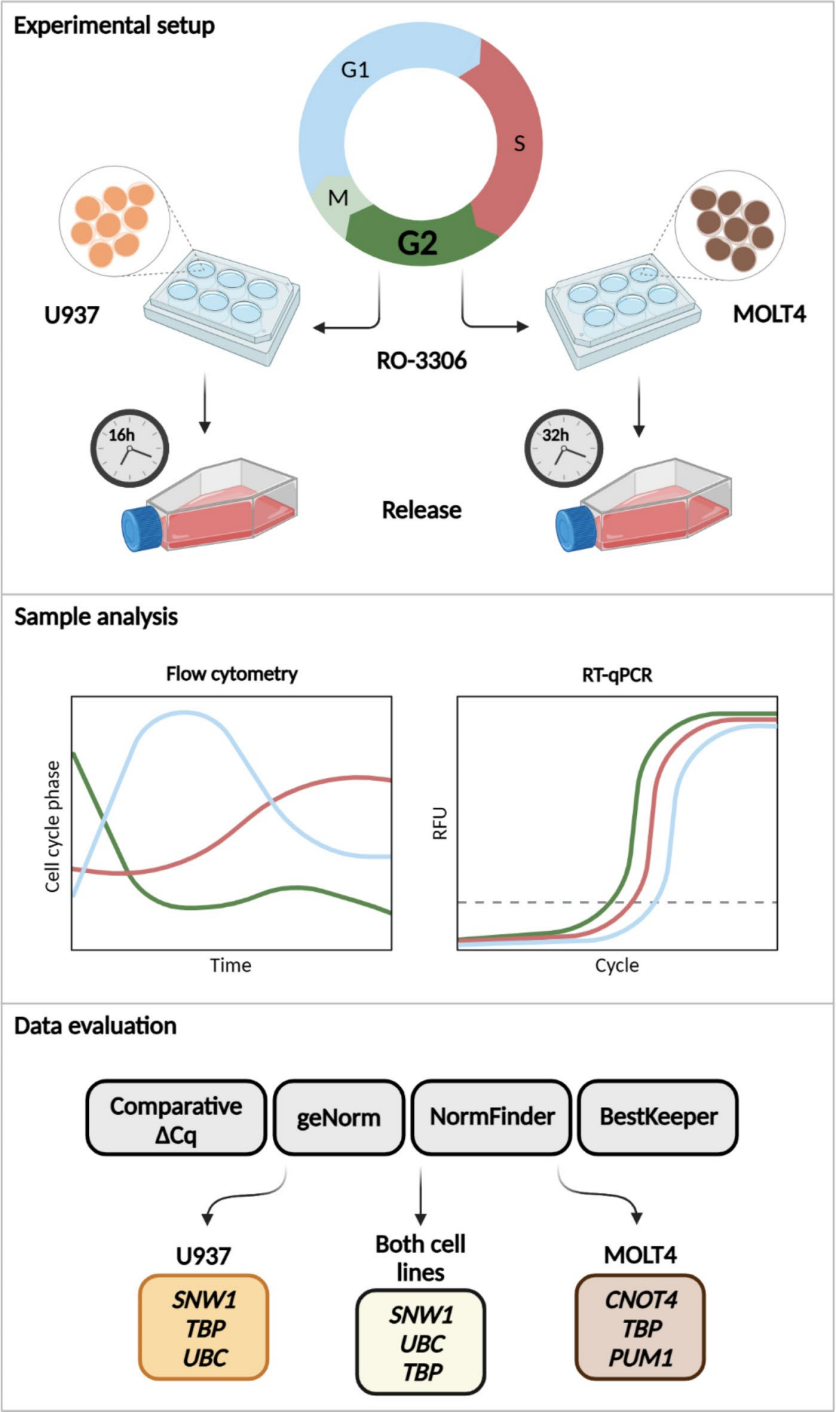
Schematic representation of the experimental design and data evaluation. Cell lines and cell cycle phases are color coded as follows: U937 cells, orange; MOLT4 cells, brown; G0/G1 phase, blue; S phase, red; G2/M phase, green. Clocks show sample collection time for each cell line. Graphs illustrate results of flow cytometry and RT-qPCR analysis. Evaluation tools are shown in grey rectangles. Top three reference genes are listed for U937 (orange), MOLT4 (brown) and for both cell lines (yellow). Created with BioRender.

Our aim was to characterize suitable reference genes for cell cycle analysis, as there are no such studies in the literature identifying reference genes with stable expression levels throughout the cell cycle. For that purpose, we selected two suspension cancer cell lines, U937 and MOLT4, which are applicable for synchronization with RO-3306 treatment. RO-3306 is a CDK1 inhibitor, which is commonly used for cell cycle arrest in G2/M phase^31^. As the generation time of U937 and MOLT4 are different, we collected U937 samples for 16 h, while MOLT4 samples for 32 h after release from RO-3306 inhibition. Each collected sample was analyzed with flow cytometry to determine cell cycle phase distribution and RT-qPCR to measure gene expression levels of the candidate reference genes (Fig. 1). Using this experimental design different phases of the cell cycle are well represented.

**Fig. 2:**
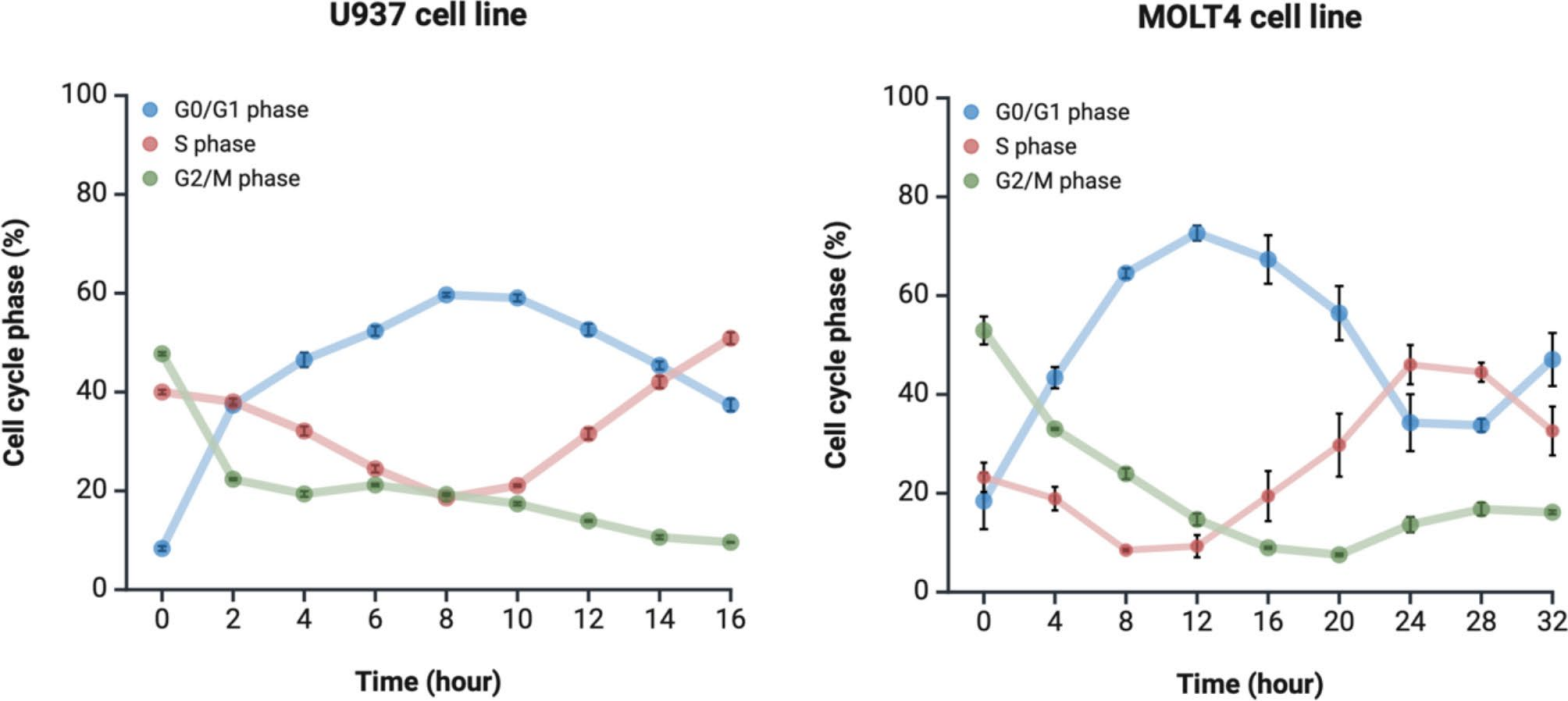
Cell cycle phase distribution for each cell line as determined using flow cytometry analysis. Percentage of cells (%) in different phases of cell cycle (G0/G1, S and G2/M) collected at given time points in the case of U937 and MOLT4 cell lines. Cell cycle phases are color coded as follows: G0/G1 phase, blue; S phase, red; G2/M phase, green. Error bars show standard error of the mean of three biological replicate samples for each time point. Note that values of several samples have low variation reflected in small, but still visible error bars. Individual graphs were created with BioRender and assembled using InkScape software.

Synchronization using RO-3306 treatment resulted in the enrichment of G2/M phase in both cell lines which was followed by the gradual accumulation of cells in G0/G1 phase, while towards the end of generation time the majority of cell population was in S phase (Fig. 2). In samples collected at 0 h after release, more than 47% of cells were in the G2/M phase in both U937 and MOLT4 cell lines. Cells in G0/G1 phase peaked at 58% at 8 h in U937 cell line, while at 71% at 12 h after release in MOLT4 cell line. S phase dominates at the end of the generation time at 16 h in U937 with 50%, while at 24 h after release in MOLT4 with 46% (Fig. 2). Cell cycle phase distribution of biological samples for both cell lines in each collected time point are summarized in Supplementary Table S1. In our experimental setup with RO-3306 CDK1 inhibition cells in G0 and G1 phase cannot be separated. Although analyzing G0 phase independently is important to investigate cells in resting state exiting the cell cycle, however, in the present study we focused on identifying reference genes in dividing cells.

Following the determination of distribution of various phases of cell cycle, RNA was isolated from samples collected at each time point. Integrity of RNA was assessed with agarose gel electrophoresis (Supplementary Fig. S2) and its purity was determined with NanoDrop (Supplementary Table S3). RNA was confirmed to be intact, enabling reverse transcription. Reverse transcribed cDNA was used for qPCR analysis to investigate the mRNA expression of 12 candidate reference gene targets (*ACTB*,* CNOT4*,* GAPDH*,* HNRNPL*,* IPO8*,* PCBP1*,* PPIA*,* PUM1*,* RPL30*,* SNW1*,* TBP*,* UBC*). For each target, three biological replicates and three technical replicates were measured. RT-qPCR measurements were carried out using the highly robust and optimized method described previously^40^. To minimize the possibility of genomic DNA contamination, primers were designed to target exon-exon junctions (intron-spanning primers) or consecutive exons separated by an intron (intron-flanking primers) whenever possible (Supplementary Table S4). Optimal annealing temperature was determined by gradient PCR for each primer pair. Product specificity was verified initially by agarose gel electrophoresis during the optimization procedure, while melting curve analysis was performed routinely after every measurement. Moreover, several other crucial parameters were assessed as well, such as determination of PCR efficiency and the linear dynamic range of reverse transcription and PCR.

Evaluation of qPCR results was performed using Comparative ΔCt, geNorm, NormFinder and BestKeeper methods. Comparative ΔCt use mean Cq values of technical replicates for evaluation and cannot consider PCR efficiency for each target. Since BestKeeper Excel based tool can only analyse a maximum of 10 genes, we used the BestKeeper tool of RefFinder, in which PCR efficiency cannot be taken into account. For analysis with geNorm raw Cq and PCR efficiency values were used. NormFinder utilizes relative expression values, which were imported from CFX Maestro software considering PCR efficiency. Using the Comparative ΔCt method, differences in Cq values for each pairwise combination of two reference genes were calculated for every biological replicate. Standard deviation (SD) of these Cq differences was determined for every gene pair, and the average SD was obtained for each target. Genes with the lowest SD values are considered suitable for data normalization^45^. GeNorm assesses the stability M values of candidate reference genes by utilizing geometric mean of SD values through all pairwise comparisons among investigated targets^46^. Unlike the other methods applied, NormFinder considers inter- and intra-group variations using expression values in each sample group^47^. Comparative ΔCt and geNorm are prone to bias toward co-regulated genes, as their rankings rely on correlations across samples. In contrast, while BestKeeper also employs correlation analysis, its ranking is mainly determined by the SD of Cq values, thus mitigating such type of bias^48^. As each evaluation method is based on different calculation strategies, the more methods are used, the more reliable the identification of stable reference genes suitable for the given experimental conditions. For quick evaluation of gene expression results without PCR efficiency values, RefFinder online tool provides all methods mentioned above for reference gene selection^49^. For more reliable analysis geometric mean of the ranking provided by the different tools can be calculated, hereby referred to as comprehensive ranking.

In case of U937 cell line *SNW1*,* TBP* and *UBC* were found to be the most stable reference genes according to Comparative ΔCt, geNorm and NormFinder, while BestKeeper suggested *RPL30*,* SNW1* and *UBC* (Table 1). Rankings of BestKeeper are mainly determined by the SD of Cq values, which reduces the likelihood of favouring co-regulated genes, thus *RPL30* is identified as the best choice. Based on our comprehensive ranking *SNW1*,* TBP* and *UBC* can be used as reliable reference genes for cell cycle analysis in U937 cells. *IPO8* and *PPIA* were found to be the least stable reference genes according to every evaluation method used. Despite that *IPO8* is considered a stable reference gene in human adipose tissues^50^, ovarian tumours^51^, lung cancer samples^52^ and between cell lines^40^, here we showed that it cannot be applied for cell cycle analysis according to our results.

In contrast, for MOLT4 cell line Comparative ΔCt and NormFinder tools proposed *CNOT4*,* TBP* and *PUM1*, while geNorm suggested *CNOT4*,* GAPDH*, and *TBP* as stable reference genes. However, BestKeeper recommended *PCBP1*,* RPL30* and *CNOT4* as the most suitable choices. Importantly, despite the changes in the rankings, *CNOT4* is present in the top three genes according to all data analysis methods. *PUM1* was identified as one of the three most stable reference genes not only across different cell lines^40^, but also throughout the cell cycle in MOLT4 cells. *ACTB* and *PPIA* were shown to be the least stable reference genes according to our comprehensive ranking. Expression of *ACTB* is proved to differ even between comparable cell types, thus its application for normalizing RT-qPCR data is disputable^53–56^. Although *UBC* was found to be stable in U937 cells, it ranked quite low in case of MOLT4 cells, underlying the differences between gene expression patterns even in these two comparable cell lines.

Regarding both cell lines together *SNW1*,* UBC* and *TBP* were identified as the top three genes in our comprehensive ranking, however all tools suggested different orders, further highlighting the differences between evaluation methods. It is worthwhile to note that stability values of the top genes identified by each evaluation method comparing the two cell lines are similar to the less stable genes identified using only one cell line showing that comparing gene expression between the two cell lines introduces more bias in the results than performing gene expression analysis throughout the cell cycle of each cell line. However, *TBP* may be suitable as it ranked second in both cell lines analyzed separately. *PPIA* and *ACTB* were found to be the least stable genes for cell cycle experiments in U937 and MOLT4. Our detailed data evaluation highlights that not all widely used reference genes are suitable for cell cycle-dependent analysis, therefore applicable reference genes must be characterized for each cell line and each experimental setup.

For reliable normalization of gene expression data, at least two reference genes are required. GeNorm suggests the optimal number of reference genes characterized with the geNorm V value, which is acceptable below 0.15. For evaluation of these 12 reference genes the V value indicated that the use of at least two reference genes is sufficient for reliable normalization in case of U937 alone, MOLT4 alone and both cell lines together as the V values were below 0.065, 0.050, and 0.0775, respectively (Supplementary Fig. S5). According to our results we suggest the investigation of at least four candidate reference genes namely *SNW1*, *CNOT4*, *TBP* and *GAPDH* for cell cycle-dependent gene expression analysis to characterize at least two reference genes with stable expression pattern. Moreover, we recommend evaluating gene expression data of candidate reference genes using different methods, available at RefFinder for quick analysis, i.e. Comparative ΔCt, geNorm, NormFinder and BestKepper. In conclusion, we propose to generate a comprehensive ranking based on the geometric mean of different rankings to identify the best choices for reliable normalization.

**Table 1:** Evaluation of candidate reference genes for U937 alone, MOLT4 alone and for both cell lines together. Comprehensive rankings are generated based on the geometric mean of ranks. SD, standard deviation. Top three genes are shown in bold.

	Comprehensive rank	Gene symbol	geNorm		Comparative ΔCt		NormFinder		BestKeeper		Geometric mean
			Rank	M value	Rank	Mean SD	Rank	Stability value	Rank	Stability value	
U937 cell line	1	***SNW1***	1	0.340	1	0.197	1	0.047	2	0.165	1.19
	2	***TBP***	2	0.354	2	0.200	2	0.077	6	0.222	2.63
	3	***UBC***	3	0.357	3	0.204	3	0.110	3	0.176	3.00
	4	*RPL30*	5	0.394	5	0.230	5	0.164	1	0.105	3.34
	5	*GAPDH*	4	0.372	4	0.215	4	0.135	5	0.208	4.23
	6	*PCBP1*	6	0.420	6	0.241	7	0.206	4	0.197	5.63
	7	*PUM1*	7	0.427	7	0.271	6	0.195	7	0.253	6.74
	8	*ACTB*	9	0.481	8	0.303	9	0.252	8	0.342	8.49
	9	*CNOT4*	8	0.474	10	0.357	8	0.249	10	0.380	8.94
	10	*HNRNPL*	10	0.527	9	0.330	10	0.309	9	0.353	9.49
	11	*PPIA*	11	0.608	11	0.400	11	0.375	11	0.539	11.00
	12	*IPO8*	12	0.681	12	0.441	12	0.423	12	0.570	12.00
MOLT4 cell line	1	***CNOT4***	1	0.248	1	0.153	3	0.075	3	0.208	1.73
	2	***TBP***	3	0.252	2	0.160	1	0.073	10	0.236	2.78
	3	***PUM1***	4	0.254	3	0.162	2	0.075	5	0.222	3.31
	4	*GAPDH*	2	0.251	5	0.180	5	0.084	4	0.212	3.76
	5	*PCBP1*	7	0.288	9	0.216	9	0.146	1	0.098	4.88
	6	*HNRNPL*	5	0.261	4	0.171	4	0.082	11	0.284	5.45
	7	*SNW1*	6	0.262	6	0.185	6	0.089	8	0.242	6.45
	8	*RPL30*	10	0.306	10	0.224	10	0.163	2	0.106	6.69
	9	*IPO8*	8	0.292	7	0.196	7	0.130	6	0.229	6.96
	10	*UBC*	9	0.293	8	0.207	8	0.136	9	0.258	8.49
	11	*ACTB*	11	0.331	11	0.234	11	0.176	7	0.238	9.82
	12	*PPIA*	12	0.644	12	0.301	12	0.428	12	0.641	12.00
Both cell lines	1	***SNW1***	1	0.421	2	0.240	3	0.113	6	0.435	2.45
	2	***UBC***	2	0.441	1	0.230	9	0.216	8	0.479	3.46
	3	***TBP***	5	0.480	4	0.267	1	0.079	10	0.628	3.76
	4	*GAPDH*	8	0.539	8	0.380	2	0.108	2	0.218	4.00
	5	*PCBP1*	3	0.473	3	0.246	6	0.176	7	0.475	4.41
	6	*RPL30*	9	0.555	9	0.393	5	0.172	1	0.138	4.49
	7	*PUM1*	4	0.475	5	0.276	4	0.145	9	0.537	5.18
	8	*CNOT4*	7	0.519	7	0.354	7	0.203	3	0.332	5.66
	9	*HNRNPL*	6	0.518	6	0.319	10	0.259	4	0.375	6.16
	10	*IPO8*	11	0.748	11	0.489	11	0.348	5	0.401	9.03
	11	*PPIA*	10	0.721	10	0.444	12	0.404	11	0.675	10.72
	12	*ACTB*	12	0.909	12	0.553	8	0.214	12	1.132	10.84

**Fig. 3:**
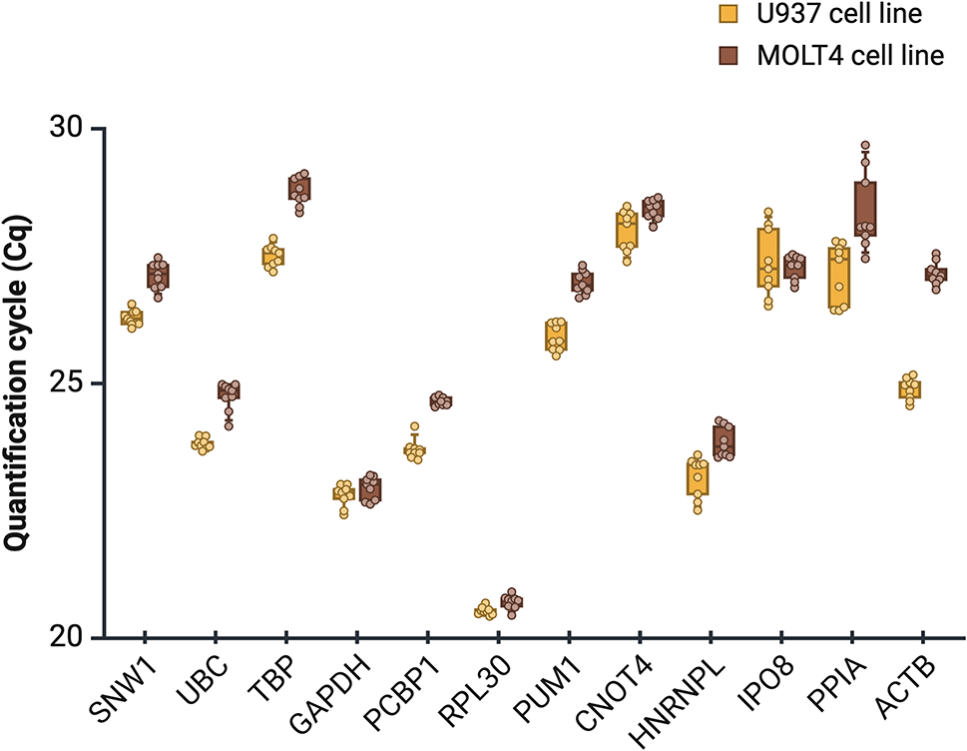
Boxplot of quantification cycle (Cq) values for biological replicates of each target for both U937 and MOLT4 cell lines. U937 and MOLT4 cell lines are colored orange and brown, respectively. The order of candidate reference genes is listed based on the comprehensive rank calculated for both cell lines together. Data between 25th and 75th percentile are represented as boxes, while whiskers show data range from 10th to 90th percentile. The average values are illustrated as lines. Graph was created with BioRender.

Cq values of candidate reference genes are presented in the order of comprehensive ranking for both cell lines investigated together (Fig. 3). Cq values and SD of *RPL30* were found to be the lowest, however, only BestKeeper ranked *RPL30* in the top three reference genes. Top six reference genes are characterized with lower SD within and between cell lines in contrast to the other six genes. Interestingly, *IPO8* was previously determined as one of the best reference gene investigating 7 normal and 13 cancer cell lines for gene expression normalization, however, here we showed that it is highly inappropriate for cell cycle-dependent analysis. Moreover, it is important to note that the commonly used *ACTB* is unsuitable both for normalization between cell lines^40,53^ and for cell cycle-dependent analysis. Previously, *CNOT4* was found to be appropriate for comparison between different cell lines and for serum starvation treatment^40^ as well as in lung cancer cells under various conditions^57^. However, regarding cell cycle analysis it is only applicable for MOLT4, but not for U937 cell line. In accordance with previous findings^20,40,57^, our comprehensive ranking indicates *SNW1* as the most stable reference gene for U937 alone and for both cell lines investigated together, thus we recommend including *SNW1* in RT-qPCR studies for reference gene selection or gene expression normalization.

## Conclusion

Our aim was to identify suitable reference genes for cell cycle-dependent gene expression analysis, as there is no such investigation addressing stable reference genes throughout the cell cycle. We established a highly robust method for cell cycle analysis in U937 and MOLT4 human leukemia cell lines using CDK1 inhibitor RO-3306 for synchronizing cells in G2/M phase followed by sample collection and determination of cell cycle phase distribution with flow cytometry. Using this method, the entire generation time was monitored, and cells enriched in the different stages of cell cycle were obtained, thus enabling the assessment of mRNA expression of 12 candidate reference genes (*ACTB*,* CNOT4*,* GAPDH*,* HNRNPL*,* IPO8*,* PCBP1*,* PPIA*,* PUM1*,* RPL30*,* SNW1*,* TBP*,* UBC*) using RT-qPCR. Measurements were carried out with our optimized RT-qPCR method described previously^40^.

For evaluation of qPCR data Comparative ΔCt, geNorm, NormFinder, and BestKeeper tools were applied, each providing different approaches for ranking gene stability. Our results showed that the most stable reference genes were *SNW1*, *TBP*, and *UBC* in U937 cells, while in MOLT4 cells *CNOT4*,* TBP* and *PUM1* were found to be the most stable reference genes during the cell cycle. Despite minor differences in rankings, *CNOT4* consistently appeared in the top three genes for MOLT4 cell line. Comprehensive rankings were determined based on the geometric mean of ranks generated by individual evaluation methods. For both cell lines *SNW1*,* TBP* and *UBC* were found to be the most stable, while *ACTB* and *PPIA* were shown to be unsuitable. Despite its common use, *ACTB* was reported inappropriate for normalization as demonstrated in several studies^40,53,54,58,59^ and here we showed that it is unsuitable for cell cycle-dependent experiments, as well. Differences in gene expression patterns between the two cell lines investigated here, underline the importance of appropriate reference gene selection for a given experimental setup and that stable reference genes must be identified separately for each cell line. Furthermore, gene expression is not only influenced by the cell line used, but also by experimental conditions, such as drug treatments, synchronization methods or stress responses. Accordingly, validating stability of intended reference genes is crucial for each experimental setup.

In this study we performed a detailed analysis of selected reference gene candidates to determine their suitability for cell cycle-dependent gene expression analysis in U937 and MOLT4 cell lines. Despite being restricted to these cell lines, our results highlight genes that may serve as more reliable options for normalization compared to commonly used unstable reference genes. We concluded that frequently used reference genes, like *ACTB*,* PPIA* and *IPO8*, are not suitable for cell cycle-dependent analysis, while *SNW1* and *CNOT4* emerged as the most stable genes for U937 and MOLT4 cell lines, respectively, making them candidates for gene expression normalization in RT-qPCR studies. For both cell lines, however, *TBP* may be suitable as it ranked second in both cell lines analyzed separately.

## Materials and methods

### Cell maintenance

U937 is an adult acute monocytic lymphoma cell line, while MOLT4 is an adult acute T lymphoblastic leukemia cell line. MOLT4 cell line was obtained from the National Cancer Institute’s Developmental Therapeutics Program (National Institutes of Health). U937 cell line was purchased from ATCC. MOLT4 and U937 cells were grown and maintained in RPMI-1640 medium with L-glutamine and sodium bicarbonate (Sigma, R8758) supplemented with 1% penicillin-streptomycin (Gibco, 15140-122) and 10% fetal bovine serum (Gibco, 10500064). All cell lines were maintained in a humidified incubator (Eppendorf, Galaxy 170R) at 37 °C with 5% CO_2_ atmosphere. The absence of mycoplasma infection was verified using genomic DNA isolation followed by PCR analysis.

### Cell synchronization

20 million cells were seeded on 6-well plates (Corning, Costar) and synchronized with 10 µM RO-3306 (Sigma, SML0569) for 20 h. After treatment, cells were centrifuged at 150 g (5810R, Eppendorf) for 5 min and washed with phosphate buffered saline solution (PBS, Sigma, P3813). Cells were resuspended in fresh medium and divided by 2 million in T25 flasks (Sarstedt). Samples were collected in every 2 (U937) and 4 h (MOLT4) for a duration of 18 and 36 h, respectively. Before each sample collection, cells were treated with 10 µM 5-ethynyl-2’-deoxyuridine (EdU) for 20 min (Click-iT™ Plus EdU Alexa Fluor™ 488 Flow Cytometry Assay Kit, Invitrogen, C10632). Cells were centrifuged at 200 g (MiniSpin, Eppendorf) for 5 min and washed with PBS. Cells were divided into two groups for RT-qPCR and for the analysis of cell cycle phase distribution with flow cytometry. Since fixed cells are required for flow cytometry analysis, RNA cannot be isolated from those samples, however, using this approach the same sample can be measured with the two different techniques.

### Determination of cell cycle phase distribution

The assay was performed using Click-iT™ Plus EdU Alexa Fluor™ 488 Flow Cytometry Assay Kit, (Invitrogen, C10632). Cells were treated with 10 µM EdU for 20 min, then collected and washed with 1% BSA (Sigma) diluted in PBS. Cells were centrifuged at 200 g for 5 min, then fixed using 4% paraformaldehyde (PFA, Sigma) diluted in PBS for 15 min. Cells were centrifuged and resuspended in 1 ml 1% BSA in PBS and stored at 4 °C until further use. Permeabilization and click-it reaction were performed according to the manufacturer’s recommendation. Attune NxT flow cytometer (Thermo Fischer Scientific) was used for the analysis of the cell cycle. The Click-iT™ Plus EdU Alexa Fluor™ 488 signal was detected with 488 nm excitation and 530/30 nm emission in the BL1 channel. Propidium iodide signal was detected with 488 nm excitation and 695/40 nm emission in the BL3 channel. Data analysis was conducted using Attune NxT software version 3.2.1.

### RT-qPCR

Cells were collected and centrifuged at 200 g for 4 min and washed with PBS. Cell pellets were lysed by vortexing the cells with 600 µl RLT buffer (RNeasy Plus Mini Kit, Qiagen) supplemented with β-mercaptoethanol and with glass beads for 1 min. Samples were stored at -20 °C until further use. For RNA isolation RNeasy Plus Mini Kit (Qiagen) was used according to the manufacturer’s recommendation. DNase treatment was also applied according to the manufacturer’s recommendation (RNase-Free DNase Set, Qiagen). Purity of the isolated RNA samples were verified using NanoDrop 2000, while their integrity were tested using 1% TBE agarose gel electrophoresis. As molecular weight size marker, GeneRuler 1 kb Plus DNA (Thermo Fischer Scientific) ladder was used. Concentration of RNA samples was measured with NanoDrop 2000. 200 ng RNA was reverse transcribed into cDNA using Maxima First Strand cDNA Synthesis Kit for RT-qPCR (Thermo Fischer Scientific) at 65 °C for 30 min. Selection of the 12 investigated candidate reference genes was discussed in detail in our previous work^40^. The qPCR measurements were performed using the optimized RT-qPCR method described previously^40^. Features of selected primers are summarized in Supplementary Table S4. For the verification of the lack of genomic DNA contamination, no reverse transcriptase (NRT) controls were used and measured randomly for 35% of samples. NRT samples were prepared for each RNA sample without using RT enzyme and buffer. For each measurement three replicates of no template controls (NTC) were used. Differences between Cq values of NRT/NTC controls and the samples were higher than 10 in most cases, and higher than 5 in all cases. Relative expression data were calculated with CFX Maestro 2.0 software (Bio-Rad), taking PCR efficiency values into account for every target. Supplementary Fig. S6 summarizes relative quantity of reference gene targets in samples collected at each time point for U937 and MOLT4 cell lines.

### Data analysis

To test the cell cycle-dependent mRNA expression of selected reference genes, Cell Cycle-Dependent Transcript Database of the Human Protein Atlas (data available at: proteinatlas.org/download/subcellular_location.tsv.zip), Cyclebase 3.0 database and targetgenereg.org dataset were assessed. Investigated candidate reference genes are either not present or have inconsistent data available about their cell cycle dependency. Investigated candidate reference genes either not present or have no data available about their cell cycle dependency. Evaluation of qPCR data was performed using the Comparative ΔCt, geNorm, NormFinder, and BestKeeper methods. Comparative ΔCt calculates the mean Cq values of technical replicates and cannot consider PCR efficiency. Comparative ΔCt calculates differences between Cq values for each pair of genes across biological replicates, and genes with the lowest SD are determined as stable. For geNorm analysis, raw Cq and PCR efficiency values were used, which evaluates the stability of reference genes using the geometric mean of SDs. For the analysis using NormFinder the required relative expression data were calculated with CFX Maestro 2.0 software (Bio-Rad), taking efficiency values into account for every target. BestKeeper online tool of RefFinder was utilized for analyzing the mean Cq values for all biological replicates, and it cannot consider PCR efficiency. Comprehensive rankings of candidate reference genes were obtained calculating the geometric mean of ranks determined by each evaluation method.

## Electronic supplementary material

Below is the link to the electronic supplementary material.

Supplementary Material 1

## Data Availability

Raw data is available upon request (e-mail: toth.otilia@ttk.hu, vertessy.beata@ttk.hu, nagy.nikolett@ttk.hu).
