# Supplementary material for: Identification of new reference genes with stable expression patterns for cell cycle experiments in human leukemia cell lines
Source: Sci Rep. 2025 Jan 7;15:1052. doi: 10.1038/s41598-024-84802-5 (PMC11707088; doi:10.1038/s41598-024-84802-5)
Supplement: Supplementary file 1 — Supplementary Material 1 [file 41598_2024_84802_MOESM1_ESM.pdf]

Supplementary information

Identification of new reference genes with stable expression patterns for cell cycle experiments in human leukemia cell lines

Otília Tóth[1,2*], Gergely Attila Rácz[2], Eszter Oláh[2,3], Máté Tóth[1], Edit Szabó[2], György Várady[2], Beáta G. Vértessy[1,2*], Nikolett Nagy[2,3*]

Affiliations

[1]Department of Applied Biotechnology and Food Science, Faculty of Chemical Technology and Biotechnology, BME Budapest University of Technology and Economics, Budapest, Hungary

[2]Institute of Molecular Life Sciences, HUN-REN Research Centre for Natural Sciences, Budapest, Hungary

[3]Doctoral School of Biology, Institute of Biology, ELTE Eötvös Loránd University, Budapest, Hungary

Correspondence and requests for materials should be addressed to Otília Tóth (e-mail: toth.otilia@ttk.hu), Nikolett Nagy (e-mail: nagy.nikolett@ttk.hu) and Beáta G. Vértessy (e-mail: vertessy.beata@ttk.hu).

Supplementary Table S1. Cell cycle phase distribution (%) at each time point after release in U937 and MOLT4 cells for three biological replicate samples.

U937 cell line				
**Time point**	**Biological replicate**	**G0/G1 phase (%)**	**S phase (%)**	**G2/M phase (%)**
**0h**	**1**	8.67	40.59	47.18
	**2**	8.95	39.12	47.65
	**3**	7.39	40.29	48.38
**2h**	**1**	36.64	39.28	21.91
	**2**	37.34	37.79	22.56
	**3**	38.10	36.96	22.58
**4h**	**1**	43.73	33.92	19.88
	**2**	48.65	31.59	18.24
	**3**	47.19	30.84	19.99
**6h**	**1**	50.44	25.88	21.56
	**2**	52.97	24.16	20.69
	**3**	53.63	23.33	21.34
**8h**	**1**	58.86	19.88	18.98
	**2**	60.15	18.21	19.01
	**3**	59.99	17.60	19.77
**10h**	**1**	59.32	21.06	17.18
	**2**	60.12	20.64	16.94
	**3**	57.68	21.48	18.05
**12h**	**1**	54.91	29.51	13.90
	**2**	51.98	31.78	14.22
	**3**	51.02	33.29	13.68
**14h**	**1**	46.48	40.21	11.32
	**2**	45.86	41.57	10.61
	**3**	43.78	44.28	9.92
**16h**	**1**	39.85	48.50	9.51
	**2**	36.23	52.15	9.52
	**3**	36.07	51.96	9.74

MOLT4 cell line				
**Time point**	**Biological replicate**	**G0/G1 phase (%)**	**S phase (%)**	**G2/M phase (%)**
**0h**	**1**	26.37	20.19	47.72
	**2**	7.37	29.16	57.45
	**3**	21.55	20.30	53.64
**4h**	**1**	46.22	16.23	33.21
	**2**	39.18	23.66	32.61
	**3**	44.76	16.85	33.22
**8h**	**1**	65.55	8.73	21.97
	**2**	62.56	8.75	25.70
	**3**	65.45	7.98	24.19
**12h**	**1**	71.01	12.50	12.54
	**2**	75.69	4.94	16.61
	**3**	71.25	10.43	14.95
**16h**	**1**	61.65	25.87	8.71
	**2**	77.08	9.42	8.83
	**3**	63.20	23.00	9.38
**20h**	**1**	48.18	38.31	7.09
	**2**	66.83	17.29	7.65
	**3**	54.33	33.64	7.92
**24h**	**1**	30.39	48.40	15.41
	**2**	45.62	38.27	10.59
	**3**	26.84	51.40	14.95
**28h**	**1**	36.06	41.28	18.50
	**2**	33.60	47.96	14.25
	**3**	31.58	44.24	17.63
**32h**	**1**	55.52	25.66	15.47
	**2**	37.11	42.16	16.27
	**3**	48.58	30.01	16.72

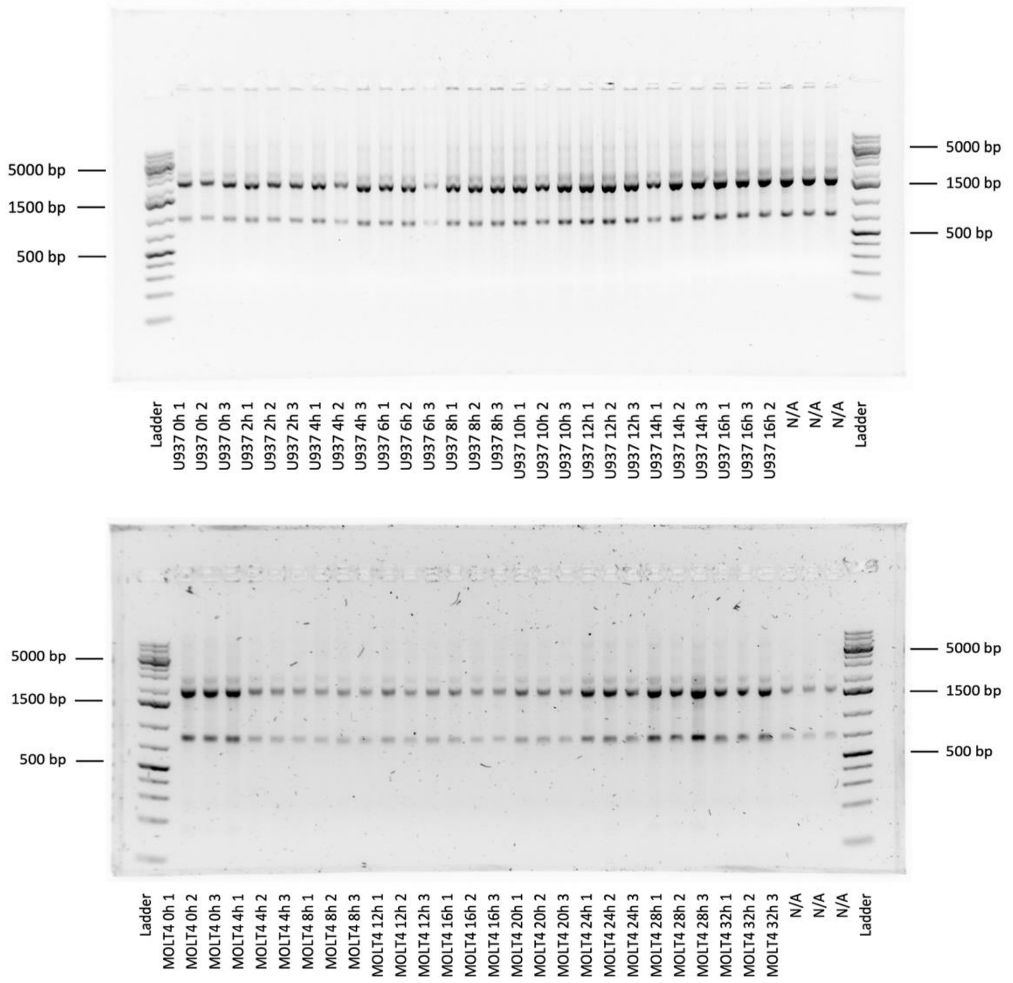

Supplementary Figure S2. Agarose gel electrophoresis of isolated RNA samples from U937 and MOLT4 cells. Image was captured by Image Lab 4.1 software (Bio-Rad) and assembled using InkScape software.

Supplementary Table S3. A260/280, A260/230 values and yield of isolated RNA samples from U937 and MOLT4 cells. Purity values and RNA concentration were measured by NanoDrop2000**.**

U937 cell line				
**Time point**	**Biological replicate**	**A260/280**	**A260/230**	**Yield (ng)**
**0h**	1	2.08	1.51	1542.0
	2	2.10	0.89	1452.0
	3	2.13	2.17	1416.0
**2h**	1	2.11	1.98	1212.0
	2	2.12	1.99	1134.0
	3	2.05	0.93	1197.0
**4h**	1	2.12	1.95	1299.0
	2	2.10	2.05	1068.0
	3	2.10	2.00	1092.0
**6h**	1	2.10	1.00	1287.0
	2	2.11	1.99	1086.0
	3	2.13	1.77	1149.0
**8h**	1	2.12	2.19	1266.0
	2	2.09	2.00	1353.0
	3	2.15	0.16	1494.0
**10h**	1	2.08	1.99	1584.0
	2	2.11	1.89	1545.0
	3	2.09	1.71	1770.0
**12h**	1	2.08	0.88	1842.0
	2	2.07	1.63	1950.0
	3	2.09	1.93	1707.0
**14h**	1	2.11	1.34	1071.0
	2	2.09	2.05	1617.0
	3	2.08	1.98	1803.0
**16h**	1	2.08	1.98	1902.0
	2	2.10	1.90	1758.0
	3	2.09	1.37	1764.0

MOLT4 cell line				
**Time point**	**Biological replicate**	**A260/280**	**A260/230**	**Yield (ng)**
**0h**	1	2.06	1.26	2280.0
	2	2.19	1.73	2604.0
	3	2.05	1.78	2976.0
**4h**	1	2.04	1.37	2739.0
	2	2.07	1.45	2661.0
	3	2.07	1.37	2724.0
**8h**	1	2.00	1.56	2523.0
	2	2.02	1.64	2568.0
	3	2.08	1.87	2757.0
**12h**	1	2.06	1.77	3588.0
	2	2.02	1.59	2895.0
	3	2.06	0.76	3009.0
**16h**	1	2.01	1.78	3603.0
	2	2.03	1.41	2694.0
	3	2.03	1.65	3687.0
**20h**	1	2.04	1.52	3903.0
	2	2.04	1.51	2898.0
	3	2.01	1.79	3681.0
**24h**	1	2.04	1.85	1611.0
	2	2.07	1.50	1692.0
	3	2.07	2.18	1356.0
**28h**	1	2.08	1.55	1533.0
	2	2.07	2.27	1794.0
	3	2.13	1.43	1014.0
**32h**	1	2.12	1.98	1128.0
	2	2.11	2.07	1161.0
	3	2.13	1.27	933.0

Supplementary Table S4. Table summarizing parameters of primers used in this study. Base pairs, bp.

Gene symbol	Primer sequences (5'-3')	Length of PCR product (bp)	Tm of PCR product (°C)	Primer design	PCR efficiency (%)
ACTB	Fw: ACAGAGCCTCGCCTTTGC<br>Rev: CGCGGCGATATCATCATCCA	76	86.9	Intron-flanking	95.2
CNOT4	Fw: GTCCAAAACCTGACTGCATGTATC<br>Rev: GGTGTTTACCCGCCTGCAT	87	80.8	Intron-spanning	96.3
GAPDH	Fw: GAGAAGGCTGGGGCTCATTT<br>Rev: TGATGACCCTTTTGGCTCCC	46	79.4	Intron-spanning	97.8
HNRNPL	Fw: CCAAGGCCTCTCTCAATGGG<br>Rev: TTCAAGCGTGTAGGCTTTGC	82	80	Intron-spanning	97.9
IPO8	Fw: GGCATACAGTTTAACCTGCCAC<br>Rev: CAGGAGAGGCATCATGTCTGTAA	118	78.6	Intron-spanning	92.5
PCBP1	Fw: ATTCGCCGGAATTGACTCCA<br>Rev: TGCCCAATAGCCTTTCACCT	49	86.4	Exonic	99.8
PPIA	Fw: TGGGTTACTTCTGAAACATCACTTGT<br>Rev: TTGACACTTCCTGGGACTGGA	85	75.1	Exonic	98.2
PUM1	Fw: TGCGGGAGATTGCTGGACAT<br>Rev: GTGTGGCACGCTCCAGTTTC	87	80.4	Intron-flanking	98.4
RPL30	Fw: TTCTCGCTAACAACTGCCCA<br>Rev: TGCCACTGTAGTGATGGACAC	90	78.4	Intron-flanking	95.9
SNW1	Fw: GCAGCTCCTGATAAGAGGTCG<br>Rev: CCGAGGATTAGGAACACCGAG	87	78	Intron-spanning	95.8
TBP	Fw: ATATAATCCCAAGCGGTTTGCTG<br>Rev: AAAATCAGTGCCGTGGTTCG	66	79.8	Intron-spanning	97.4
UBC	Fw: GGTCGCAGTTCTTGTTTGTGG<br>Rev: TTCACGAAGATCTGCATTGTCAAG	60	78.4	Exonic	100.7

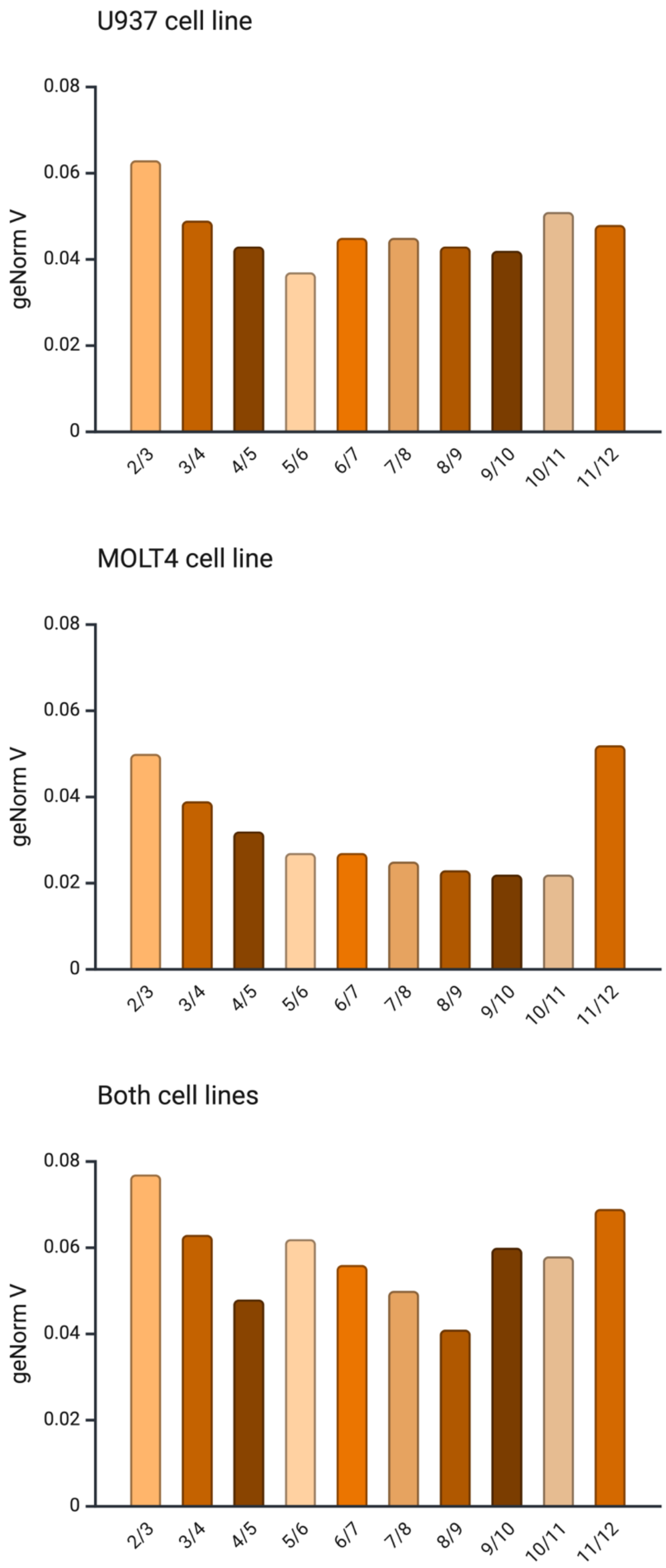

Supplementary Figure S5. Bar charts representing geNorm V values for U937 alone, MOLT4 alone and both cell lines together, respectively. V value below 0,15 indicates the appropriate number of reference genes for normalization. Scale of Y axis uniformly shows geNorm V values from 0 to 0,08, indicating that two reference genes are sufficient for normalization in every case. Created with BioRender.

U937 cell line

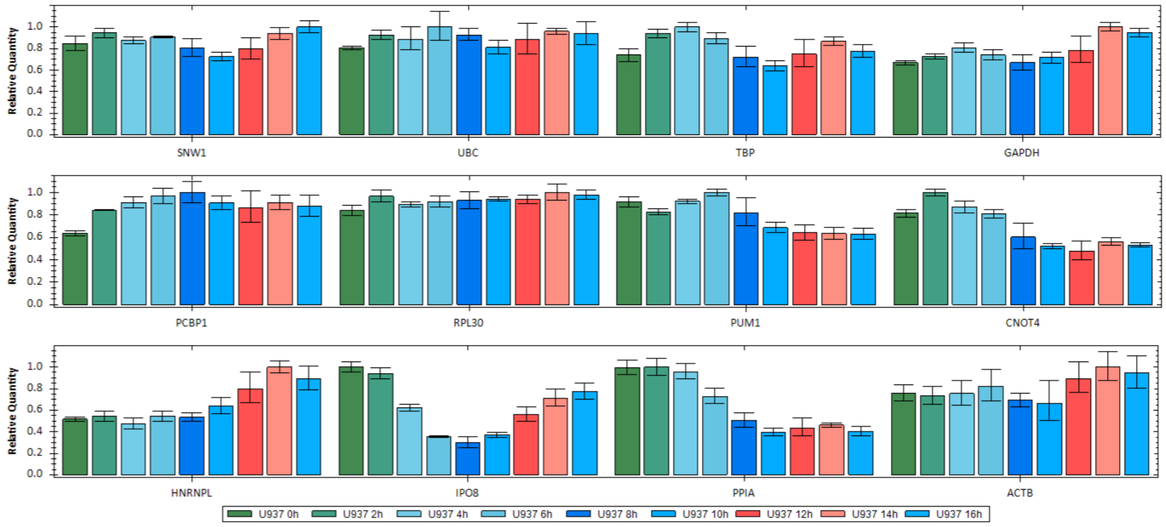

MOLT4 cell line

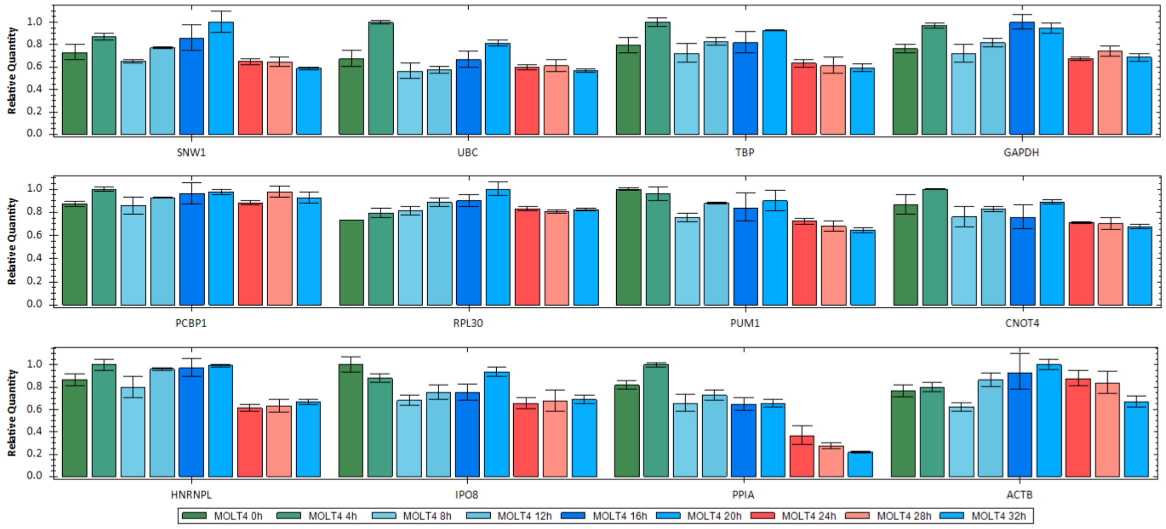

Supplementary Figure S6. Relative quantity of reference gene targets in samples collected at each time point for U937 and MOLT4 cell lines. Error bars represent standard error of the mean. Relative quantity values were calculated, and bar charts were illustrated by BioRad CFX Maestro software. Image was assembled by InkScape software.
